# Effects of combined low-dose caffeine and taurine on anaerobic, neuromuscular, and cognitive performance: a double-blind randomized placebo-controlled crossover study

**DOI:** 10.1186/s13102-026-01863-x

**Published:** 2026-07-08

**Authors:** Yakup Köse, Mehmet Ulaş, Emrah Atay

**Affiliations:** Faculty of Sport Science, Burdur Mehmet Akif Ersoy University, Burdur, Türkiye

**Keywords:** Caffeine, Taurine, Wingate, Neuromuscular performance, Cognitive performance

## Abstract

This study investigated the acute effects of low-dose caffeine (2 mg·kg⁻^1^) and taurine (10 mg·kg⁻^1^), administered alone and in combination, on anaerobic, neuromuscular, and cognitive performance in trained male athletes. Thirty-two participants, each with at least two years of consistent resistance training (≥ 3 sessions·week⁻^1^), completed experimental trials using a double-blind, randomized, placebo-controlled crossover design following ingestion of caffeine, taurine, a caffeine–taurine combination, or placebo. Combined caffeine and taurine supplementation resulted in higher handgrip strength values compared to placebo [*p* = 0.044; d = 0.51, small effect] and greater average power than caffeine alone [*p* = 0.006; d = 0.64, moderate effect] and placebo [*p* = 0.001; d = 0.91, moderate effect]. Taurine supplementation alone did not significantly affect performance outcomes. No statistically significant differences were observed among conditions for peak power, cognitive performance, ratings of perceived exertion, or blood lactate concentrations (*p* > 0.05). Overall, these findings suggest that co-ingestion of low-dose caffeine and taurine may provide modest improvements in selected neuromuscular and anaerobic performance outcomes without affecting cognitive, perceptual, or metabolic responses.

**Trial registration**

ClinicalTrials.gov, NCT07466654, registered on 06/03/2026. Retrospectively registered.

**Supplementary Information:**

The online version contains supplementary material available at https://doi.org/10.1186/s13102-026-01863-x.

## Introduction

Athletes have consistently pursued nutritional strategies to improve performance. Given that marginal improvements can affect outcomes in elite competitions, bioactive compounds that support both physical and mental performance have gained prominence in sports nutrition. These compounds are present in everyday foods as well as in dietary supplements [45, 50].

Caffeine (CAF; 1,3,7-trimethylxanthine) is one of the most widely used ergogenic aids among athletes [75]. As the most commonly consumed pharmacological and psychoactive substance after water, caffeine is notable for its potential effects on physical performance, attention, alertness, and cognitive function [84]. The performance-enhancing properties of caffeine are primarily attributed to both central and peripheral physiological mechanisms. Centrally, caffeine increases neurotransmitter release by antagonizing adenosine A1, A2A, and A2B receptors, which leads to heightened alertness and reduced perceived effort [1, 16]. Peripherally, caffeine supports neuromuscular function and improves excitation–contraction coupling by increasing Na⁺/K⁺-ATPase activity and enhancing intracellular Ca^2^⁺ bioavailability [37, 54]. Caffeine (CAF) intake is typically classified into three dose categories: low (≤ 3 mg/kg), medium (3–6 mg/kg), and high (≥ 9 mg/kg) [69]. This classification highlights that caffeine’s effects on performance are dose-dependent and guides the selection of appropriate intake strategies based on exercise characteristics. Evidence indicates that CAF consumption at 2–9 mg/kg enhances interval sprint performance, muscle endurance, and time-trial performance, and acutely improves muscle endurance, strength, power, speed, and jumping ability in both aerobic and anaerobic activities [32, 36]. Recent investigations into very high CAF doses (9–13 mg/kg) suggest potential ergogenic effects at these levels [26, 60, 81]. However, such high doses are rarely practical or sustainable and are frequently associated with increased side effects. For example, administration of 12 mg/kg CAF resulted in adverse effects, including tachycardia, palpitations, anxiety, nervousness, headache, and insomnia in most participants [26]. Moderate doses, such as 6 mg/kg, have also been linked to side effects like tremor, elevated heart rate, headache, gastrointestinal disturbances, muscle pain, and cognitive impairment [3, 31]. While similar ergogenic benefits have been observed across studies, side-effect profiles are less consistently reported [11, 59]. Conversely, low-dose CAF intake (≤ 3 mg/kg) can yield comparable psychological and physical performance benefits, though individual responses vary more widely and the risk of side effects is lower [69]. Current literature generally recommends CAF doses of 3–6 mg/kg body mass to optimize ergogenic outcomes. Due to CAF’s rapid absorption, the timing (30–90 min before exercise) and form of intake (pill, liquid, or gum) appear to have limited influence on performance. Nevertheless, the optimal supplementation protocol may depend on exercise type and duration, individual caffeine tolerance, and muscle contraction characteristics [36, 71].

Taurine (2-aminoethanesulfonic acid) is a sulfur-containing amino acid considered conditionally essential in humans and non-human primates [66]. Synthesized through cysteine metabolism, taurine is among the most abundant free amino acids in mammalian tissues, comprising approximately 50–60% of the total free amino acid pool [41, 64, 68]. High concentrations of taurine are found in skeletal muscle tissue, with intracellular levels ranging from approximately 5 to 20 µmol/g in excitable tissues, including skeletal muscle, heart, and brain [19]. Nutritionally, taurine is a natural component of most meat and seafood and is also present in commonly consumed energy drinks [51, 70]. Physiologically, taurine participates in several fundamental biological processes, including osmoregulation, inhibition of protein phosphorylation, interaction with ion channels, membrane stabilization, and regulation of calcium homeostasis [39, 41, 65, 67]. Once taken up by muscle cells, taurine enhances muscle contraction efficiency by promoting Ca^2^⁺ handling in the sarcoplasmic reticulum, thereby contributing to the regulation of muscle function [22, 38]. Taurine’s regulation of calcium homeostasis is associated with increased muscle strength and endurance, as it enhances the function of calcium-binding proteins during muscle contraction [24, 27, 72, 79]. Additionally, taurine has been reported to reduce cellular damage in muscle disorders through its antioxidant effects and may facilitate recovery of muscle function [19, 73]. Following ingestion, plasma taurine levels rise within approximately 10 min, peak at 0.03–0.06 mmol/L within 60 min, and return to baseline after about 6.5 h [29].

Recent investigations have examined the effects of taurine (TAU) intake on various components of exercise performance. Despite extensive research, inconsistencies persist in the literature regarding the ergogenic potential of taurine supplementation, and the topic remains a subject of scientific debate. Some studies have found no significant impact of taurine intake on sports performance [28, 54], whereas others have reported positive effects on specific performance parameters [78, 83]. Taurine has been studied extensively, particularly in relation to aerobic endurance and high-intensity sprint exercises [10, 48, 77]. A meta-analysis of 15 randomized controlled trials indicated that taurine intake of 1–6 g may enhance jumping performance and prolong time to exhaustion during exercise [9]. However, the precise physiological mechanisms by which taurine influences exercise performance remain unclear, complicating the interpretation of findings across varying doses and exercise intensities [48]. Additionally, research on taurine combined with other ergogenic substances has demonstrated that taurine intake with caffeine can increase peak and mean power output during repeated Wingate tests compared to placebo [57, 78]. Collectively, these findings suggest that taurine's effects on performance are context-dependent and may vary depending on the method of administration.

Although previous studies have examined the effects of caffeine and taurine on exercise performance in both isolated and combined forms, comprehensive investigations into their combined effects, particularly at low doses, on anaerobic, neuromuscular, and cognitive performance remain scarce. Much of the existing research addresses only a single performance domain or assesses these substances in isolation, which limits the ability to identify potential combined effects between caffeine and taurine. Additionally, differences in dosage protocols and methodological approaches hinder comparability across studies. Therefore, to address this gap in the literature, the present study aimed to evaluate the effects of isolated and combined low-dose caffeine and taurine supplementation on anaerobic performance, neuromuscular responses, cognitive function, perceived exertion, and blood lactate responses in trained men using a controlled experimental design. It was hypothesized that combined low-dose caffeine and taurine supplementation would lead to greater improvements in performance-related outcomes than either isolated supplementation or placebo.

## Materials and methods

### Participants

Thirty-two male participants satisfied the inclusion criteria, which specified engagement in a minimum of three strength-training sessions per week for at least two years. Their mean age was 19.40 ± 1.88 years; mean height, 181.00 ± 9.75 cm; mean weight, 79.51 ± 12.17 kg; mean body fat, 14.63 ± 4.42%; and mean skeletal muscle mass, 37.47 ± 6.70 kg (see Table 1). Participants were recruited between October 15 and November 15, 2025, and all experimental sessions were completed during this period.

**Table 1:** Demographic characteristics of the participants

Variable	Unit	Mean ± SD
Age	years	19.40 ± 1.88
Height	cm	181.00 ± 9.75
Body weight	kg	79.51 ± 12.17
Body fat percentage	%	14.63 ± 4.42
Skeletal muscle weight	kg	37.47 ± 6.70

The inclusion criteria for the study were as follows: 1) Participants were male, aged 18 to 25 years; 2) engaged in strength training at least three days per week for the past 24 months; and 3) free from injuries to skeletal muscles, joints, or tendons in the past two years. Participants were also instructed to abstain from physical activity, caffeine, and alcohol for 24 h before each test, to record all food and beverages consumed, and to maintain a consistent diet for the 24 h preceding the next test.

The minimum sample size for our study was determined using G-Power software (University of Düsseldorf, Düsseldorf, Germany) [25]. This analysis utilized a priori and F tests to calculate statistical power based on the design of our study, which involved repeated measures (within factors) ANOVA. The power analysis was conducted using *G*Power* (version 3.1), with the following parameters: significance level (α) = 0.05, minimum effect size (f) = 0.25, number of measurements = 4, and desired power (1—β) = 0.90. The results indicated that a minimum of 30 participants was required to achieve an actual power of 90.3%. These parameters were carefully selected based on standards established in similar studies in the literature, ensuring that our sample size is adequate for generating reliable results.

### Experimental design

**Fig. 1:**
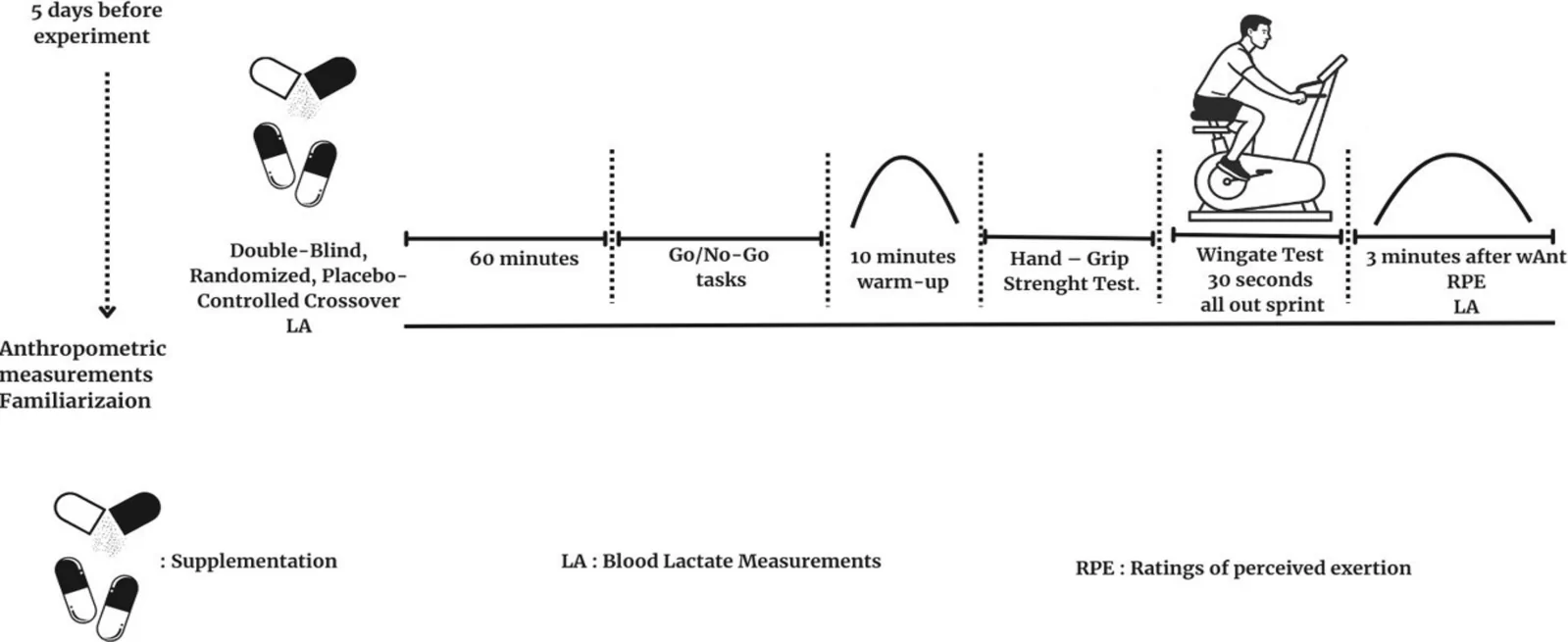
Experimental design

This study employed a double-blind, placebo-controlled, crossover experimental design. Participants completed a total of five sessions. In the first session, anthropometric measurements were taken without any reinforcement, and the performance test protocols were explained and administered for familiarization purposes. This session aimed to acquaint participants with the tests, minimize learning effects, and enhance test–retest reliability. Following this, four experimental sessions were conducted. Each participant was exposed to four different reinforcement conditions—caffeine (2 mg/kg) plus maltodextrin (300 mg) (CAF + PLA), taurine (10 mg/kg) plus maltodextrin (300 mg) (TAU + PLA), caffeine (2 mg/kg) plus taurine (10 mg/kg) (CAF + TAU), and maltodextrin (300 mg) only (PLA + PLA)—in random order. Participants were assigned to a balanced, randomly generated set of all *4!* (24) possible condition orders to ensure equal initial exposure to each condition. Randomization was performed using a custom-built balanced permutation algorithm powered by https://www.randomizer.org. This method aimed to minimize the risk of systematic errors related to the order of conditions. Neither the participants nor the researchers knew which supplement was administered, thereby maintaining double-blind conditions. To prevent supplement effects from being influenced by physiological variations due to circadian rhythms, all experimental sessions were conducted within 72 h and at the same time interval for each participant (2:00 PM–4:00 PM, ± 30 min). The handgrip strength test, 30-s Wingate anaerobic power test, Go/No-Go cognitive test, Rating of Perceived Exertion, and blood lactate levels were measured during each session. All tests were administered in a fixed order within a controlled laboratory environment maintained at a constant temperature of 24 °C to ensure data comparability. Participants were monitored throughout the study for any potential adverse effects related to supplementation. The study was conducted in the Athlete Performance Measurement and Analysis Laboratory at the Faculty of Sports Sciences, Burdur Mehmet Akif Ersoy University, Türkiye. The laboratory temperature was continuously monitored using the VZN Medical Device Hydrothermograph, which has a measuring range of − 50 to + 70 °C and an accuracy of ± 1 °C, confirming that ambient conditions complied with international quality and safety standards (see Fig. 1).

### Supplementation protocol

Doses were determined by weighing on a balance (TN-series; maximum capacity: 50 g, readability: 0.001 g) with a precision of 1 mg. All supplements were prepared in powder form. The compounds were encapsulated in indistinguishable opaque gelatin capsules and administered orally with tap water. Capsules contained 300 mg of maltodextrin in the placebo (PLA) condition, and either 2 mg/kg caffeine (CAF), 10 mg/kg taurine (TAU), or a combination of 2 mg/kg CAF and 10 mg/kg TAU in the other conditions.

All supplements were supplied by the same manufacturer (My Protein, Manchester, UK) and administered to participants 60 min before the start of the experimental sessions. This timing was selected to ensure comparability with previous studies and to correspond with the peak plasma levels of CAF [37] and TAU [42]. The CAF dose was set at the lower end of the range recommended by the International Society of Sports Nutrition [37]. The TAU dose was chosen because it reflects the lower end of doses reported in recent meta-analyses [9, 12] and corresponds to amounts commonly found in commercial energy drinks [47]. 72 h before each experimental condition, participants were instructed to follow dietary guidelines established by dietitians to ensure consistent energy intake (65% carbohydrate, 20% fat, 15% protein). This period was implemented to standardize nutritional intake and recovery status between experimental sessions. Participants were instructed to record all food and beverage intake throughout the dietary control period to promote consistency between sessions; however, dietary compliance was based on self-report and was not objectively verified through nutritional or caloric analysis. During the final 24 h before testing, participants were instructed to avoid caffeine-, taurine-, and alcohol-containing products to minimize potential acute carryover effects.

### Blinding

To maintain the study's double-blind conditions, neither the participants nor the researchers responsible for data collection and performance testing were aware of the supplement administered during each experimental session. All supplements were prepared in identical opaque gelatin capsules by an independent researcher who was not involved in participant testing, data collection, or statistical analysis. The capsules were coded prior to the experimental sessions, and the allocation code remained confidential until data analysis was complete. This procedure ensured that both participants and investigators remained blinded to the supplementation conditions throughout the study.

### Body composition measurements

Participants’ stature was assessed to the nearest 0.1 cm using a precision stadiometer (Holtain Ltd., Crymych, UK). Measurements were conducted with participants standing upright, barefoot, and aligned according to the Frankfurt horizontal plane to maintain consistency. Body composition was evaluated using a segmental multi-frequency bioelectrical impedance device (InBody 270, Biospace Co., Ltd., Seoul, Korea), which performs impedance assessments at frequencies of 20 and 100 kHz across five body segments (right and left arms, trunk, right and left legs). The procedure, which lasted approximately 15 s, required participants to stand barefoot on the device’s foot electrodes while gripping the hand electrodes, according to the manufacturer’s standardized instructions. The measurement provided key outputs including total body mass, skeletal muscle mass, and body fat percentage.

### Go/No Go task

To assess cognitive performance, participants completed the Go/No-Go Test, which measures attention and impulse control by requiring responses to specific stimuli (Go condition) and withholding responses to others (No-Go condition). Participants were instructed to respond as quickly and accurately as possible throughout the task. Cognitive performance was evaluated by analyzing reaction times and correct and incorrect response rates. The test was administered in a laboratory setting using PEBL (Psychology Experiment Building Language), a widely used software for psychological experimental design and cognitive testing. The task consisted of 40 trials in total, with a 3:1 Go-to-No-Go ratio (75% Go and 25% No-Go trials) and an inter-stimulus interval of 1500 ms. Each participant completed the test 60 min after supplementation intake. The administration time for each session was approximately 2–3 min, and the testing procedure was conducted under standardized conditions for all participants. No participants were excluded due to low task accuracy (< 60% correct) [7, 55].

### Hand-grip strength test

Maximal voluntary handgrip strength was measured using a digital handgrip dynamometer (Takei Physical Fitness Test, model T.K.K. 5401 Grip-D, range 5–100 kg; accuracy ± 2.0 kg). Participants were instructed to exert maximal isometric grip force with their dominant hand in response to a verbal command from the researchers. Each participant completed two trials, separated by a one-minute rest period to minimize muscle fatigue, and the highest value was recorded for analysis.

### Wingate anaerobic performance test

The Wingate anaerobic performance test was administered using a friction-braked cycle ergometer (Monark Ergomedic 894E, Monark, Stockholm, Sweden) integrated with a microcomputer system. Prior to the test, participants completed a standardized warm-up designed to prepare the musculature and enhance test reliability. This warm-up consisted of 5 min of cycling at a cadence of 70 revolutions per minute (rpm) against a fixed resistance of 60 W. To stimulate anaerobic metabolism and activate neuromuscular pathways, participants performed two 5-s maximal sprints without resistance during the 2nd and 3rd minutes of the warm-up. Following a brief rest period, the primary Wingate test was initiated in accordance with the procedure described in [5]. Participants performed the 30-s maximal effort test against a braking force equivalent to 7.5% of their body weight (0.075 kg/kg body mass). The test commenced with a low-resistance "rolling start," the weight basket was held in place, and participants pedaled at 50 rpm. Once the cadence reached 130 rpm, the resistance load was automatically applied. From this point, participants were instructed to sustain maximal pedaling effort throughout the 30-s test duration, accompanied by consistent verbal encouragement. All performance data were collected via the Monark Anaerobic Test Software (Version 3.3.0.0) and transferred to the computer system for analysis. Parameters obtained included Peak Power (W), Relative Peak Power (W/kg), Mean Power (W), Relative Mean Power (W/kg), Minimum Power Output (W), and the Fatigue Index, calculated as FI = [(Peak Power − Minimum Power)/Peak Power] × 100.

### Blood lactate level measurements

This study assessed blood lactate levels using the Lactate Scout 4 analyzer (EKF Diagnostics, Cardiff, UK), a portable device based on electrochemical detection technology. The analyzer requires only 0.5 µL of capillary blood per test and can measure lactate concentrations within a range of 0.5 to 25 mmol/L. It provides rapid and accurate results, with a measurement error margin limited to ± 0.2 mmol/L, and completes each reading in under 10 s. The device also features automatic calibration and integrated quality control via test strips, ensuring the reliability and validity of the measurements.

Capillary blood samples were obtained from the participants’ earlobes at two specific time points: (1) before the exercise to determine baseline lactate levels, and (2) at the 3rd minute post-exercise to capture peak lactate concentrations. These time points were selected to evaluate the acute metabolic response to maximal anaerobic exertion.

### Rating of perceived exertion

The Borg Rating of Perceived Exertion (RPE) Scale was utilized in this study to assess participants’ subjective perception of effort following high-intensity anaerobic exercise. This psychophysiological scale, which ranges from 6 to 20, enables individuals to rate how strenuous the exercise felt, with higher values reflecting greater perceived exertion. The inclusion of the Borg RPE scale allowed for the evaluation of internal responses elicited by the Wingate anaerobic test under four experimental conditions: low-dose caffeine, taurine, their combination, and placebo. When interpreted in conjunction with blood lactate measurements, these perceptual responses provided valuable insight into both physiological and psychological reactions to supramaximal exertion, thereby offering a more holistic understanding of the effects of each supplementation strategy.

### Statistical analysis

Statistical analyses were performed using JASP software (version 0.19.3). A one-way repeated measures ANOVA assessed differences among the four experimental conditions within the same group. The Shapiro–Wilk test evaluated the normality assumption, which is suitable for small to moderate sample sizes (*p* > 0.05 indicating normal distribution). Mauchly’s test of sphericity assessed the sphericity assumption required for repeated measures ANOVA. When this assumption was violated, the Greenhouse–Geisser correction was applied, and degrees of freedom were adjusted. For variables with statistically significant main effects in the ANOVA, post hoc pairwise comparisons were conducted. The Bonferroni correction was used to control the risk of Type I error due to multiple comparisons. This analytic strategy adhered to recommended guidelines for statistical reporting in studies utilizing JASP software [73, 76]. The magnitude of differences among the four experimental conditions—caffeine, taurine, their combination, and placebo—was quantified using Cohen’s d effect size and 95% confidence intervals. Effect sizes were interpreted according to the classification values < 0.2 were considered negligible, 0.2–0.6 as small, > 0.6–1.2 as moderate, > 1.2–2.0 as large, and > 2.0 as very large. Statistical significance was set at *p* < 0.05 [40].

This study was reported in accordance with the CONSORT 2025 (Consolidated Standards of Reporting Trials) guidelines [85].

## Results

### GO/NO GO task results

The study analyzed cognitive performance results under mean accuracy and go no go response time.

**Fig. 2:**
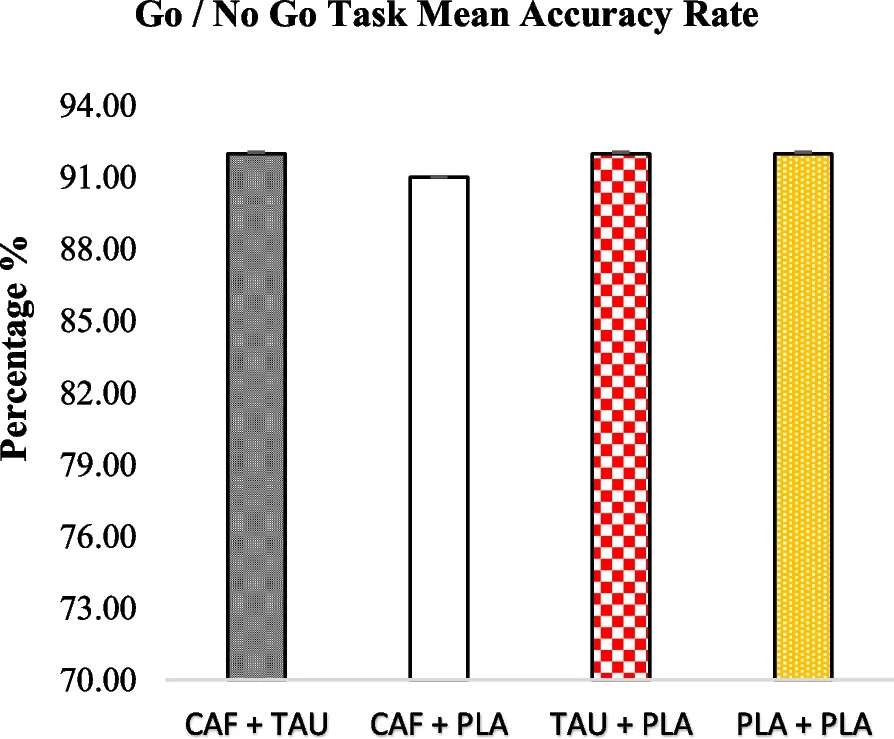
Go/No Go task mean accuracy rate. Error bars represent standard deviation. CAF + TAU: Caffeine + Taurine Intake. CAF + PLA: Caffeine + Placebo Intake. TAU + PLA: Taurine + Placebo Intake. PLA + PLA: Plasebo + Placebo Intake

Mean accuracy rates on the Go/No Go Task did not differ significantly across the four different intake conditions [F_(3,93)_ = 1.202, *p* = 0.313, n^2^p: 0.037] (see Fig. 2 and Supplementary Table S1).

**Fig. 3:**
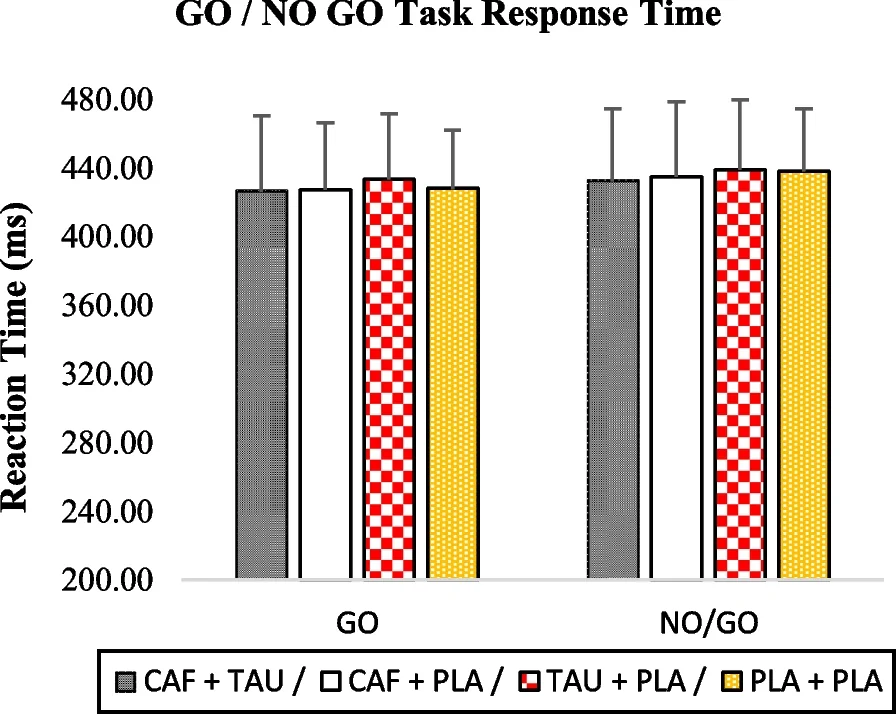
Go/No Go task response time. Error bars represent standard deviation. CAF + TAU: Caffeine + Taurine Intake. CAF + PLA: Caffeine + Placebo Intake. TAU + PLA: Taurine + Placebo Intake. PLA + PLA: Plasebo + Placebo Intake

Similarly, no significant differences in response times were observed across the four different intake conditions for the Go task [F(_3, 93_) = 0.42, *p* = 0.73, η^2^p = 0.013] and the NoGo task [F(_3, 93_) = 0.41, *p* = 0.72, η^2^p = 0.013] (see Fig. 3 and Supplementary Table S1).

Additionally, post hoc power analyses for the non-significant cognitive outcomes of the Go/No-Go test (mean accurate, Go reaction time, and No-Go reaction performance) indicated limited statistical power to detect negligible effects.

### Hand-grip strength test results

**Fig. 4:**
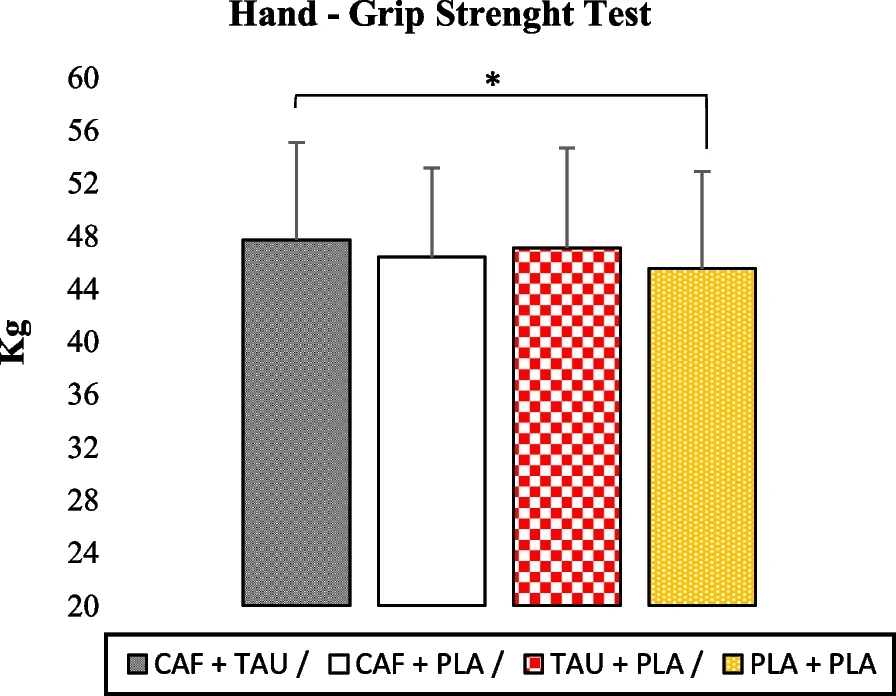
Hand—grip strength test. Error bars represent standard deviation. *: *p* < 0.05: Statistically significant difference in favor of Caffeine + Taurine Intake. CAF + TAU: Caffeine + Taurine Intake. CAF + PLA: Caffeine + Placebo Intake. TAU + PLA: Taurine + Placebo Intake. PLA + PLA: Plasebo + Placebo Intake

In the analysis of hand grip strength results, a statistically significant omnibus effect was observed among the four intake conditions [F_(3, 93)_ = 3.346, *p* = 0.026, η^2^p = 0.097] (see Fig. 4 and Supplementary Table S1).

Post-hoc analyses indicated that hand grip strength values in the CAF + TAU condition were higher compared to the PLA + PLA condition (*p* = 0.044; 95% CI = 0.04–4.28; Cohen's d = 0.51, small effect). However, this difference did not reach statistical significance after Bonferroni correction for multiple comparisons (Bonferroni-corrected α = 0.05/6 = 0.0083).

### Wingate anaerobic performance test results

The Wingate anaerobic performance test analysis provides valuable insights into peak power (PPO, W·kg⁻^1^) and average power (APO, W·kg⁻^1^).

**Fig. 5:**
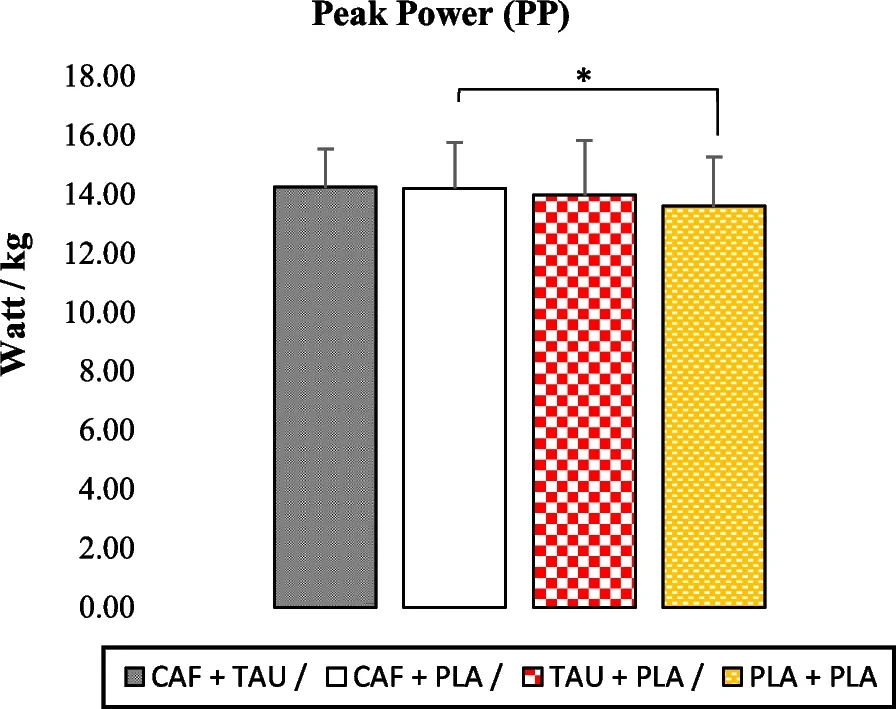
Peak Power Watt/Kg. Error bars represent standard deviation. *: *p* < 0.05: Statistically significant difference in favor of Caffeine + Plasebo Intake. CAF + TAU: Caffeine + Taurine Intake. CAF + PLA: Caffeine + Placebo Intake. TAU + PLA: Taurine + Placebo Intake. PLA + PLA: Plasebo + Placebo Intake

**Fig. 6:**
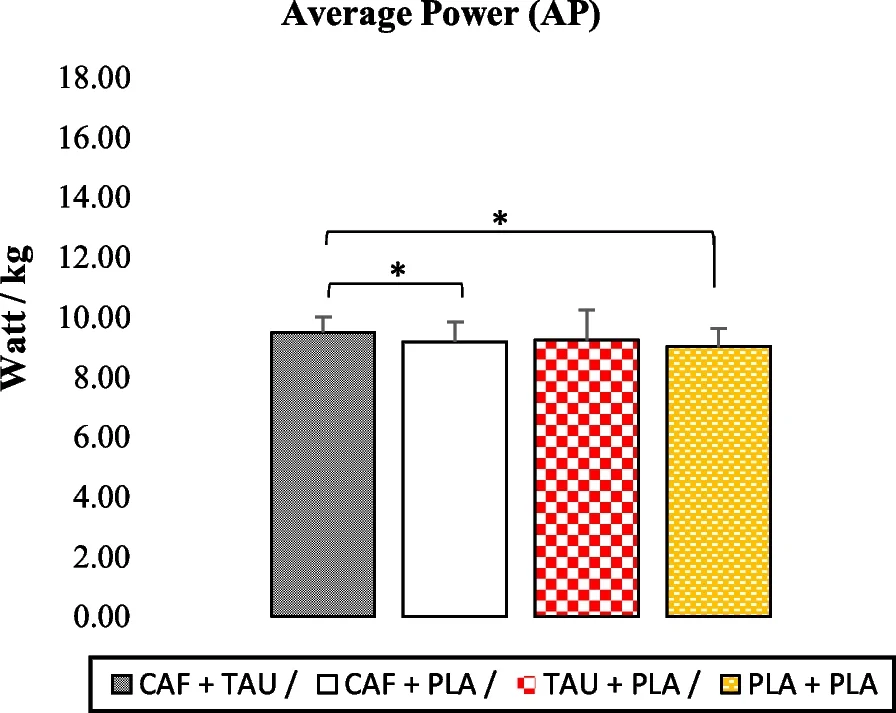
Average Power Watt/Kg. Error bars represent standard deviation. *: *p* < 0.05: Statistically significant difference in favor of Caffeine + Taurine Intake. CAF + TAU: Caffeine + Taurine Intake. CAF + PLA: Caffeine + Placebo Intake. TAU + PLA: Taurine + Placebo Intake. PLA + PLA: Plasebo + Placebo Intake

The results indicated no statistically significant difference in peak power among the four intake conditions [F_(3, 93)_ = 2.783, *p* = 0.057, η^2^p = 0.082] (see Fig. 5 and Supplementary Table S1). In contrast, a statistically significant difference in average power was observed across the four intake conditions [F_(3, 93)_ = 5.636, *p* = 0.007, η^2^p = 0.154] (see Fig. 6 and Supplementary Table S1).

Post-hoc analyses revealed that the CAF + TAU combination demonstrated significantly higher average power values than both the CAF + PLA (*p* = 0.006; 95% CI = 0.06–0.55; Cohen's d = 0.64, moderate effect) and PLA + PLA (*p* = 0.001; 95% CI = 0.22–0.75; Cohen's d = 0.91, moderate effect) conditions.

### Blood lactate level results

**Fig. 7:**
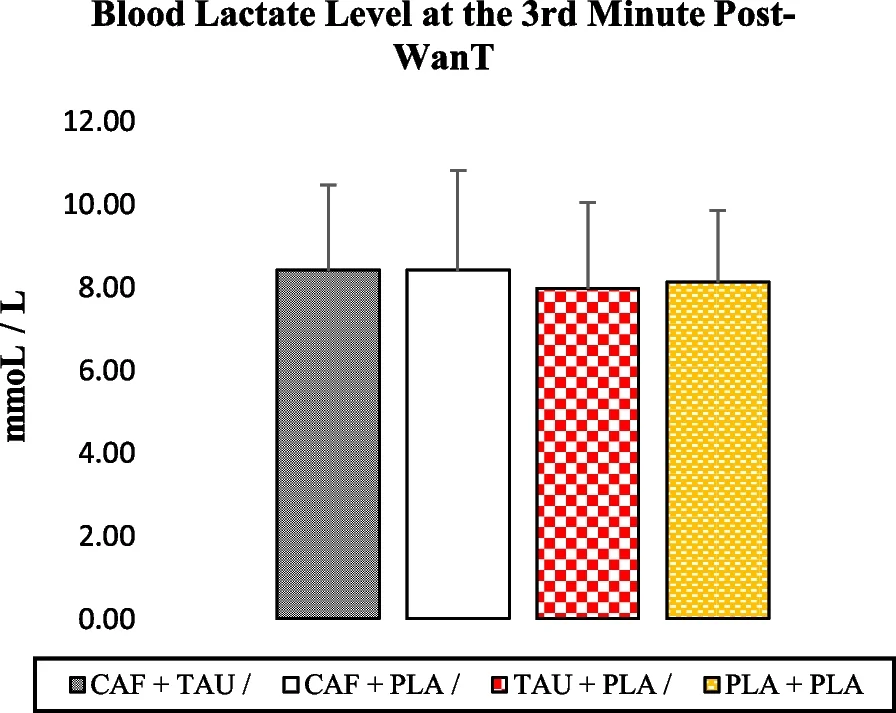
Blood lactate level at the 3rd minute post-WanT. Error bars represent standard deviation. CAF + TAU: Caffeine + Taurine Intake. CAF + PLA: Caffeine + Placebo Intake. TAU + PLA: Taurine + Placebo Intake. PLA + PLA: Plasebo + Placebo Intake

According to the blood lactate level results, no statistically significant difference was determined among the four different intake conditions in terms of blood lactate values ​​measured at the 3rd minute after the Wingate test [F_(3, 93)_ = 0.436, *p* = 0.693, η^2^ₚ = 0.014] (see Fig. 7 and Supplementary Table S1).

### Rating of perceived exertion results

**Fig. 8:**
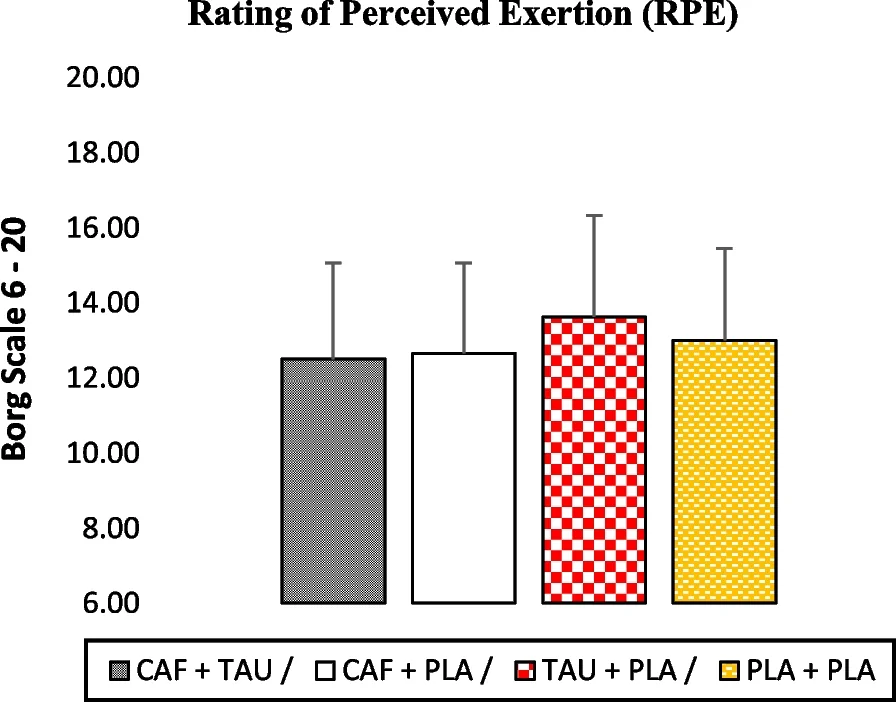
Rating of Perceived Exertion (RPE). Error bars represent standard deviation. CAF + TAU: Caffeine + Taurine Intake. CAF + PLA: Caffeine + Placebo Intake. TAU + PLA: Taurine + Placebo Intake. PLA + PLA: Plasebo + Placebo Intake

Analysis of Rating of Perceived Exertion (RPE) scores showed no statistically significant difference among the four different intakes [F_(3, 93)_ = 1.930, *p* = 0.137, η^2^p = 0.059] (see Fig. 8 and Supplementary Table S1).

## Discussion

This study investigated the acute effects of low-dose caffeine (2 mg/kg) and taurine (10 mg/kg) supplementation, both alone and in combination, on anaerobic performance, neuromuscular performance, and cognitive performance in trained male athletes who had been training regularly at least three days a week for the past two years. The findings showed that combined caffeine and taurine intake was associated with modest improvements in neuromuscular performance compared to the placebo condition. Regarding anaerobic performance outputs, caffeine intake resulted in higher peak power (W/kg) than placebo, while the combination of caffeine and taurine showed a greater increase in average power (W/kg) than either caffeine alone and placebo. However, no significant differences were found between intake conditions in cognitive performance (Go/No-Go task), RPE, and blood lactate levels. One of the most remarkable aspects of this study is that it is among the first to simultaneously examine the acute effects of a low-dose caffeine and taurine combination on anaerobic, neuromuscular, and cognitive performance in trained male athletes. The literature includes only a limited number of studies examining the effects of combined caffeine and taurine intake on performance outcomes, particularly those evaluating anaerobic, neuromuscular, and cognitive performance simultaneously.

A randomized, double-blind study was conducted with 20 elite male boxers to investigate the effects of 6 mg/kg caffeine, 3 g taurine, and placebo on Wingate anaerobic and cognitive performance across four experimental conditions [57]. The study reported statistically significant improvements in peak power (PP, W/kg), mean power (AP, W), ratings of perceived exertion (RPE), and cognitive performance with the caffeine and taurine combination. However, no significant difference in blood lactate levels was found between the intake conditions. The caffeine dose in this study was about three times higher than that in the current study. The absolute amount of taurine was also significantly higher. These differences may have contributed to the differences in performance responses between the two studies. Additionally, the absence of significant effects on cognitive performance should be interpreted cautiously, as the relatively short duration of the Go/No-Go task (approximately 2–3 min) may have limited the paradigm's sensitivity to detect subtle caffeine-induced cognitive changes. In another study [78] examining the effects of caffeine and taurine combinations on anaerobic performance, evaluated the effects of caffeine (5 mg/kg), taurine (50 mg/kg), placebo, and caffeine + taurine combination intake on the Wingate anaerobic performance test in seven male athletes using a single-blind, crossover design. The study results indicated that all intake conditions resulted in significant increases in peak power (PP) and mean power (AP) values ​​compared to placebo. While these findings are similar to current study results in terms of mean power outputs in both combined intake and caffeine intake, the limited effect of taurine intake on the WanT may be related to methodological factors such as the relatively small sample size used in the study, the relatively high doses administered, and the single-blind design. Ergogenic effects identified in studies involving male athletes cannot be directly generalized to female athletes due to physiological differences, including body size, composition, hormonal profiles, and oral contraceptive use [52]. While the number of relevant studies remains limited, existing literature includes investigations of combined caffeine and taurine supplementation in female athletes. For instance [44], compared the effects of 6 mg/kg caffeine (CAF), 1 g taurine (TAU), placebo (PLA), and CAF + TAU on Wingate anaerobic performance test outcomes in 17 female athletes accustomed to caffeine, employing a randomized, double-blind, placebo-controlled crossover design. The study reported that combined CAF + TAU intake significantly improved peak power (PP, Watt) and mean power (AP, Watt) compared to other conditions. However, no statistically significant differences were observed in blood lactate concentrations and ratings of perceived exertion (RPE). Within this framework, evidence regarding the ergogenic effects of combined caffeine and taurine supplementation is supported by findings from both individual experimental studies and synthesis-level analyses. From a broader methodological perspective, a systematic review and network meta-analysis were conducted to comprehensively assess the effects of combined caffeine and taurine intake on physical performance, cognitive function, and physiological responses [20]. The outcomes of the meta-analytic assessment demonstrated that combined supplementation exerted more pronounced effects than either isolated caffeine or taurine intake, particularly on high-intensity performance parameters and cognitive outcomes, such as anaerobic capacity and reaction time. In contrast, alterations in perceived exertion were more strongly associated with caffeine administered alone, whereas blood lactate responses appeared to differ depending on whether caffeine or taurine was consumed. These findings may indicate a potential combined influence of caffeine and taurine on performance and cognitive responses; however, the underlying mechanisms remain unclear, and physiological responses may vary depending on the context and the specific component. Although some evidence suggests possible interactive effects of combined caffeine and taurine intake on performance, studies examining these components in isolation remain important to better understand the extent of each component’s contribution to the observed responses.

In the present study, the increase in peak power (PP) during the WanT test following isolated caffeine (CAF; 2 mg/kg) suggests that this dose may be sufficient to support short-term maximal power production. However, the lack of significant changes in average power (AP), neuromuscular performance (handgrip strength), cognitive performance (go/no-go task), perceived difficulty level (RPE), and blood lactate levels suggests that this dose may have been insufficient to produce a broader ergogenic effect through central and peripheral mechanisms. Caffeine's effects on performance are dose-dependent, especially true in protocols with a high glycolytic load, such as the WanT test. The literature shows heterogeneity in findings regarding the effects of acute isolated caffeine intake on WanT performance. In this context, no significant changes in WanT performance or blood lactate levels were reported following 6 mg/kg caffeine intake [33]. Similarly, neither 5 mg/kg caffeine supplementation [14, 49] nor a lower dose of 3 mg/kg [28] significantly improved WanT performance. In contrast, a randomized, double-blind study found that 5 mg/kg of caffeine enhanced WanT performance without affecting perceived difficulty [21]. Moreover, a meta-analysis encompassing 16 studies examining isolated caffeine doses ranging from 3 to 6 mg/kg demonstrated that this dosage range generally resulted in significant improvements in peak and mean power outputs during the WanT [34]. In another study by the same author, which considered caffeine habit, it was reported that 3 mg/kg of isolated caffeine did not increase WanT performance [35]. Interestingly, the vast majority of studies in the literature reporting improvements in WanT performance, both peak and average power, used caffeine doses higher than the 2 mg/kg dose applied in the present study. This suggests that low-dose caffeine intake may be adequate for short-term maximal power output, but may have more limited effects on parameters related to sustained performance and total work output. Previous research involving both male and female athletes indicates that the effects of isolated caffeine intake on anaerobic performance and physiological responses are limited and context-dependent. These findings suggest that the potential ergogenic effects of caffeine should be evaluated using both physical and cognitive performance measures. In the present study, isolated caffeine intake did not produce a statistically significant effect on cognitive performance indicators. This result aligns with studies reporting limited or variable effects of acute caffeine intake [13, 46, 62], although some research suggests that low-dose caffeine intake (approximately 1 mg/kg) may enhance cognitive functions such as attention, alertness, and reaction time [80, 82]. Nevertheless, most of these studies have been conducted in non-athlete populations, and evidence regarding the effects of low-dose caffeine on both cognitive and physical performance in athletes remains limited. Empirical evidence demonstrates that higher doses of caffeine improve cognitive performance. For example, a 5 mg/kg caffeine dose significantly increases response accuracy in the flanker task among male participants [21], while in female athletes with low habitual caffeine consumption, both 3 mg/kg and 6 mg/kg doses of caffeinated coffee significantly improved reaction time and response accuracy compared with placebo [43]. Other studies have observed comparable stimulant effects across diverse cognitive tasks and experimental paradigms [2, 23, 43, 56, 57, 74]. Collectively, these findings indicate that the cognitive effects of caffeine depend on dose, task type, participant characteristics, and habituation.

The present study found that low-dose isolated taurine intake did not produce statistically significant effects on WanT performance, neuromuscular performance, cognitive performance, perceived exertion (RPE), or blood lactate levels. Previous research suggests that taurine may have only a limited impact on performance during short-duration, high-intensity anaerobic exercises such as the WanT [17, 53]. Nevertheless, the literature demonstrates substantial heterogeneity in reported dose–response relationships. Improvements in WanT performance have been observed following taurine supplementation at doses of 3 g in elite male boxers [57], 4.3 g in male team-sport athletes during repeated WanT protocols [78], and 6 g in male speed skaters [10]. In contrast, lower taurine doses appear to be less effective, as no significant changes in WanT performance were reported following 1 g taurine intake in female athletes [44] or 2 g taurine supplementation in sub-elite female rugby players [8]. However, within the same study, higher taurine doses of 4 g and 6 g were associated with significant improvements in WanT performance [8]. Collectively, these findings suggest that the ergogenic effects of taurine may be less apparent when administered at relatively low doses, and that performance responses may be influenced by supplementation dosage. Comparable results have been observed in studies involving anaerobic activities other than the WanT. For example, research has shown that 6 g of taurine intake did not enhance 400 m swimming performance in male swimmers [6, 15, 17], suggesting that the ergogenic effects of taurine may depend on exercise type, duration, and the predominant energy system. Therefore, the use of a low-dose taurine protocol in the present study, which is below the dose ranges reported in studies demonstrating ergogenic effects, explains the limited performance responses observed.

The literature examining the effects of taurine supplementation on blood lactate concentrations and perceived exertion (RPE) similarly demonstrates a heterogeneous pattern. Evidence indicates that taurine intake at doses of 1 g [44], 2 g [8], 3 g [17, 57] and 6 g [15] does not result in significant alterations in blood lactate levels, findings consistent with those of the present study. In contrast, other investigations have reported dose-related increases in blood lactate concentrations following taurine supplementation, with elevations observed after 3 g [6] and 6 g [10] intake compared with placebo. In terms of perceived exertion (RPE), taurine supplementation at doses of 1 g [4, 44], 1.6 g [63], and 3 g [18, 57] has been reported to have no significant effect. In contrast, a limited number of studies have indicated that higher-dose taurine supplementation, including 6 g [10] and 50 mg·kg⁻¹ [58], is associated with reductions in RPE. These discrepancies are likely attributable to methodological differences, such as the administered taurine dose, participants' training status and gender, exercise protocols, duration of taurine intake, and timing of measurements. Notably, low-dose taurine supplementation may not provide sufficient physiological stimulation via central and peripheral mechanisms, resulting in ambiguous performance and perceptual outcomes. The absence of a significant effect of low-dose taurine intake on cognitive performance in the present study should be interpreted in the context of the limited literature addressing this parameter at lower supplementation doses. Evidence indicates that 1 g of taurine intake does not significantly affect cognitive performance [61]. In contrast, studies employing higher taurine doses have reported divergent findings, suggesting a potential dose-dependent effect on cognitive performance. For instance, taurine intakes of 2 g [30] and 3 g [57] have been associated with improvements in specific cognitive performance measures. However, the small number of studies investigating the acute effects of taurine intake on cognitive performance in athletes limits the ability to generalize these findings and to establish a definitive dose–response relationship.

Overall, the present findings demonstrated that co-ingestion of caffeine and taurine was associated with modest improvements in neuromuscular performance compared with placebo. Regarding anaerobic performance, caffeine supplementation increased peak power compared with placebo, whereas co-ingestion of caffeine and taurine increased average power compared with both caffeine alone and placebo. However, the observed improvements, particularly the greater average power response following co-ingestion of caffeine and taurine, may have been influenced, at least in part, by inter-individual differences in caffeine responsiveness. Because habitual caffeine consumption, caffeine tolerance, and long-term intake patterns were not quantified, it is not possible to determine the extent to which these factors contributed to the observed variability in ergogenic responses. Therefore, although the present findings indicate beneficial effects of caffeine supplementation and the co-ingestion of caffeine and taurine on specific performance outcomes, the magnitude and practical relevance of these effects should be interpreted with appropriate caution.

Several factors should be considered when interpreting the findings of the present study. First, the circulating bioavailability of caffeine and taurine was not directly measured; therefore, the precise degree of physiological exposure resulting from the supplementation protocol could not be determined for each participant. Additionally, inter-individual differences in absorption, metabolism, and distribution rates may have influenced the timing and magnitude of performance responses. In addition, although participants received standardized dietary instructions and were asked to avoid caffeine-rich products before each experimental session, adherence was based on self-compliance and was not objectively or biochemically verified. Food intake records were not formally analyzed, and the effectiveness of blinding was not assessed. These factors may have introduced additional variability and should therefore be considered when interpreting the present findings. Furthermore, habitual caffeine consumption was not quantified, which represents an important methodological limitation, as regular caffeine intake may alter responsiveness to supplementation through tolerance-related mechanisms. Although short-term dietary intake was standardized and participants were instructed to avoid caffeine-rich foods before each experimental session, long-term habitual caffeine and taurine consumption patterns were not assessed or controlled, and participants were not stratified by caffeine use. This is important because chronic intake patterns may influence tolerance and central nervous system–mediated ergogenic responses, particularly when relatively low doses are administered. Finally, the absence of direct neurophysiological and metabolic measurements limits the ability to explain the mechanisms underlying the observed performance changes. Therefore, future studies should combine performance assessments with pharmacokinetic measurements, quantify habitual caffeine intake, classify participants according to caffeine consumption status, and include relevant neurophysiological and metabolic biomarkers to provide a broader understanding of the isolated and combined effects of caffeine and taurine across different doses and intake timings.

## Conclusion

In summary, this study demonstrates that acute low-dose supplementation with caffeine and taurine produces distinct effects on performance, contingent upon whether the compounds are ingested individually or in combination. The combined caffeine and taurine condition produced greater improvements in neuromuscular performance than placebo, whereas caffeine alone resulted in higher PP during anaerobic assessments. Additionally, co-ingestion of caffeine and taurine improved AP more than either caffeine alone or a placebo. In contrast, taurine supplementation alone did not significantly influence anaerobic or neuromuscular performance outcomes. These results suggest that, even at low doses, caffeine and taurine may interact to influence specific acute performance responses, particularly those related to neuromuscular performance.

Despite these performance-related effects, no significant differences emerged between supplementation conditions in cognitive performance, perceived exertion, or blood lactate responses. This finding suggests that the ergogenic effects of low-dose caffeine and taurine supplementation may be specific to certain neuromuscular and anaerobic performance parameters rather than indicative of a generalized physiological response. Future research should include female athletes to investigate potential sex-specific dose–response relationships and physiological differences that may affect responsiveness to caffeine and taurine. Additionally, incorporating chronotype assessments and systematically examining time-of-day effects may yield a more comprehensive understanding of how low-dose caffeine and taurine supplementation interact with circadian rhythms to influence performance.

## Supplementary Information

Supplementary Material 1.

Supplementary Material 2.

Supplementary Material 3.

## Data Availability

All data supporting the findings of this study are available in the Open Science Framework (OSF) repository: (https://osf.io/a8bg9/overview).
